# Natural ingredients in the regulation of abnormal lipid peroxidation: a potential therapy for pulmonary diseases

**DOI:** 10.3389/fphar.2024.1507194

**Published:** 2024-12-20

**Authors:** Yundou Liu, Chunyi Wang, Mengru Li, Yi Zhu, Ke Liu, Yufei Liu, Maocai Luo, Chuantao Zhang

**Affiliations:** Department of Respiratory Medicine, Hospital of Chengdu University of Traditional Chinese Medicine, Chengdu, China

**Keywords:** lipid peroxidation, oxidative stress, pulmonary diseases, natural ingredients, mechanism

## Abstract

Pulmonary diseases are a major category of diseases that pose a threat to human health. The most common drugs currently used to treat lung diseases are still chemical drugs, but this may lead to drug resistance and damage to healthy organs in the body. Therefore, developing new drugs is an urgent task. Lipid peroxidation is caused by the disruption of redox homeostasis, accumulation of reactive oxygen species (ROS), depletion of glutathione (GSH), and inactivation of glutathione peroxidase 4 (GPX4). Lipid peroxidation is closely related to the occurrence and progression of respiratory diseases, including acute lung injury, asthma, pulmonary fibrosis, pulmonary hypertension, chronic obstructive pulmonary disease, and lung cancer. Natural ingredients have high safety, high availability, and low cost, and can regulate lipid peroxidation through multiple pathways and targets, making them valuable new drugs. This article aims to summarize the pharmacology and mechanism of natural ingredients targeting lipid peroxidation in the treatment of lung diseases. The reviewed data indicate that natural ingredients are a promising anti-lipid peroxidation drug, mainly alleviating lipid peroxidation through the cystine/glutamate antiporter (System X_c_^−^)/GSH/GPX4 axis, Nrf2 pathway, and ROS pathway. In the future, it will still be necessary to further study the mechanisms of natural products in treating pulmonary diseases through lipid peroxidation and conduct multi-center, large-sample clinical trials to promote the development of new drugs.

## 1 Introduction

Pulmonary diseases are a large group of diseases that endanger human health, mainly including acute lung injury (ALI), asthma, chronic obstructive pulmonary disease (COPD), pulmonary hypertension, pulmonary fibrosis, and lung cancer. Chronic pulmonary disease is one of five non-communicable disease areas that contribute to the highest mortality and morbidity globally (Soriano et al., 2020). Statistically, asthma which is a part of chronic pulmonary disease, affected an estimated 262 million people in 2019 and caused 455,000 deaths (Wang et al., 2023b). COPD is the third leading cause of death globally, causing around 3 million deaths annually which also poses a serious risk to life and health (Venkatesan, 2024). Lung cancer is a significant public health concern, and it was the most frequently diagnosed cancer in 2022, which was also the leading cause of cancer death, with an estimated 1.8 million deaths (Bray et al., 2024). However, the current pharmacological treatment of respiratory diseases still fails to meet the existing needs, and the development of novel drugs is an urgent task nowadays (Cheong et al., 2020).

Current research indicates that oxidative stress is a significant pathogenesis for respiratory diseases (Luo et al., 2023). Under normal circumstances, low concentrations of oxygen free radicals in lung tissue participate in resisting exogenous pathogens and immune function (Junod, 1989). However, when lung tissue undergoes pathological changes and generates a large amount of oxygen free radicals due to physical conditions, oxygen free radicals become an important factor in the damage of lung tissue cells in the body. Oxygen free radicals have an initiating effect on the membrane lipid peroxidation chain reaction, which can alter membrane permeability and fluidity, produce lipid peroxides, and cause lipid metabolism disorders (Ryrfeldt et al., 1993). In recent years, lipid peroxidation has been discussed in atherosclerosis, brain tumors, gynecological, obstetric diseases, and lung diseases (Gianazza et al., 2020; Jaganjac et al., 2021; Lu et al., 2024). A study indicates that the parameters of lipid peroxidation malondialdehyde (MDA) were significantly associated with variables reflecting lipid disturbances. In lung cancer patients, parameters related to lipid alterations are associated with oxidative stress (Zabłocka-Słowińska et al., 2019). In COPD patients, increased levels of lipid peroxidation and its final product 4-hydroxynonenal (4-HNE) can be detected, indicating a correlation between lipid peroxidation and the pathogenesis of COPD (Lakshmi et al., 2020). In addition, lipid peroxidation also plays an important role in the development of pulmonary fibrosis. Increased concentrations of lipid peroxidation products, oxidized proteins, and an altered antioxidant enzyme status have often been reported in epithelial lining fluid of idiopathic pulmonary fibrosis patients (Cameli et al., 2020). Moreover, the study indicates that the lungs may be more resistant to the initiation and/or spread of lipid peroxidation processes than the liver (Schweich et al., 1994). Therefore, lipid peroxidation, as an important process of oxidative stress, plays a crucial role in the occurrence and development of lung diseases (Gęgotek et al., 2016). Treating pulmonary diseases by regulating lipid peroxidation is a potential therapeutic tool.

A study indicates that anti-lipid peroxidation drugs may be divided into three categories: ①Inhibiting enzymatic lipid peroxidation by inhibiting enzyme activation or reaction, such as fullerenols (Chen et al., 2024b). ②Inhibition of free radical mediated-lipid peroxidation may be achieved by inhibiting chain initiation, chain propagation, and/or chain termination, including carotenoids. ③Lipid peroxidation induced by singlet oxygen may be inhibited by the inhibition of singlet oxygen formation, including Vitamin E and Vitamin C (Niki et al., 2005). However, the types of drugs on the market that can regulate lipid peroxidation to treat lung diseases are still limited, making it difficult to meet the growing medical demand. In this context, natural ingredients, as an untapped treasure trove of new drug resources in nature, provide unprecedented possibilities for the development of novel anti-lipid peroxidation drugs. Numerous studies have shown that botanical drugs contain abundant bioactive components that exhibit significant regulatory effects on lipid peroxidation processes. For example, chlorogenic acid can enhance the activity of antioxidant enzymes, thereby reducing lipid peroxidation levels and ultimately delaying paraquat-induced lung injury and fibrosis progression (Larki-Harchegani et al., 2023). Ellagic acid can improve lipid peroxidation in elastase induced emphysema model in rat, also achieved by enhancing the activity of antioxidant enzymes (Mansouri et al., 2020). Therefore, natural ingredients are gradually receiving widespread attention from researchers and the medical community in the treatment of lung diseases (Lalsangpuii et al., 2024). Natural ingredients not only provide new molecular frameworks and mechanisms of action for new drug development, but may also bring therapeutic options with fewer side effects and higher efficacy, which is of great significance for improving the quality of life and prognosis of patients with lung diseases. This paper summarises the current status of natural ingredients for the treatment of pulmonary diseases through lipid peroxidation, with the purpose of providing new insights into the understanding of current research and the future direction of natural ingredients in pulmonary diseases.

## 2 Materials and methods

An online literature search was carried out at PubMed, covering 2014 until September 2024. The following keywords were used: “lipid peroxidation” and “natural ingredients”, or “active ingredient”, or “herbal medicines”, or “Chinese herbal”, and “acute lung injury”, or “asthma”, or “pulmonary fibrosis”, or “pulmonary arterial hypertension”, or “COPD”, or “lung cancer”. The references of all retrieved articles were also reviewed to include relevant literature.

## 3 Mechanisms and hazards of lipid peroxidation

### 3.1 Lipid peroxidation

**FIGURE 1:**
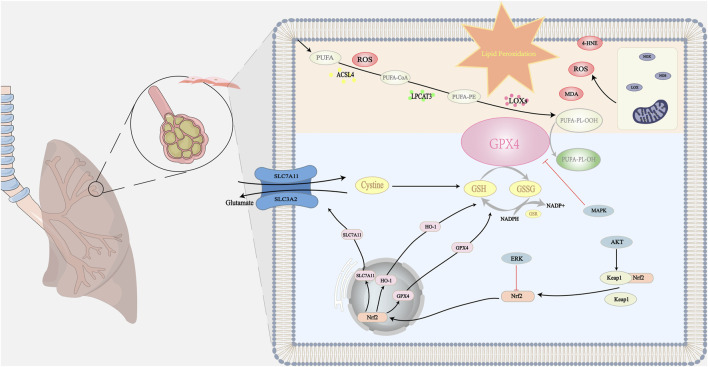
The mechanism of lipid peroxidation: When lipid peroxidation occurs, polyunsaturated fatty acids in phospholipid membranes generate lipid hydroperoxides under the action of various enzymes. This process is accompanied by the formation of many secondary products, including MDA, 4-HNE, and ROS. These products can act as oxidizing agents with toxic effects on cells. Natural ingredients can protect cells from toxic effects by directly or indirectly scavenging lipid peroxidation products. The antioxidant system in the organism is mainly through GPX4-related pathways. Natural ingredients stimulate the downstream target HO-1 and enhance SLC7A11 protein expression by upregulating Nrf2 gene expression. Thus, GPX4 is directly or indirectly activated to inhibit lipid peroxidation by converting lipid hydroperoxides to nontoxic lipids and alcohols. In addition, multiple targets are involved in regulating the SLC7A11/GPX4 axis, including activation of the System X_c_^−^, which promotes GSH synthesis and GPX4 activation to regulate lipid peroxidation. At the same time, natural ingredients can reduce the inhibitory effect on GPX4 by inhibiting certain signaling pathways such as MAPK, which ultimately inhibits lipid peroxidation. Symbols: Black arrow (↓): indicates promotion. The bold red arrow (⊥): indicates inhibition.

Lipid peroxidation is a process under which oxidants such as free radicals or non-radical species attack lipids containing carbon-carbon double bond(s), especially polyunsaturated fatty acids (PUFA) (Rochette et al., 2022). This process is influenced by oxidative stress: Reactive oxygen species (ROS) is a key component in mediating lipid peroxidation (Wang et al., 2023a). Substances produced by oxidative stress can directly oxidize membrane lipids, resulting in membrane lipid peroxidation (Pope and Dixon, 2023). The increase in hydrogen peroxide (H_2_O_2_) production and iron release from proteins in oxidative stress by superoxide ion (O_2_^·−^) and peroxynitrite (ONOO^−^) causes a marked elevation in the production of lipid peroxidation products, including 4-hydroxy-2-nonenal (Forman and Zhang, 2021). Lipid peroxidation is broadly divided into three steps. Initiation: a promoter such as a hydroxyl radical extracts allyl hydrogen to form a carbon-centric lipid radical. Propagation: lipid radicals react rapidly with oxygen to form lipid peroxy radicals, which extract hydrogen from another lipid molecule to produce new lipid radicals (continuous chain reaction) and lipid hydrogen peroxide. Termination: antioxidants such as vitamin E provide hydrogen atoms to lipid peroxy radicals to form the corresponding vitamin E radicals, which react with another lipid peroxy radical to form non-free radical products (Wang et al., 2023a). From the point of view of enzymatic reactions, PUFA-CoA is generated by acyl coenzyme A synthetase long-chain family member 4 (ACSL4) catalyzes the ligation of free PUFA (such as arachidonic acid and adrenoic acid) to CoA to generate PUFA-CoA. PUFA-CoA subsequently binds to phosphatidylethanolamine (PE) to form PUFA-PE catalyzed by lysophosphatidylcholine acyltransferase 3 (LPCAT3). PUFA-PE is susceptible to lipoxygenase (LOX)-mediated free radical-induced oxidation forming the peroxidation product PUFA-PL-OOH (Doll et al., 2017; Stoyanovsky et al., 2019; Qiu et al., 2024). Figure 1 illustrates the mechanism of lipid peroxidation.

A direct product of lipid peroxidation is lipid hydroperoxides (Gaschler and Stockwell, 2017), as their hydroperoxyyl chains become more hydrophilic and tend to bind to the lipid-water interface, resulting in changes in membrane permeability and fluidity (Balakrishnan and Kenworthy, 2024). The secondary products of lipid peroxidation include isoglutaraldehyde, MDA, 4-HNE (Valgimigli, 2023). These secondary products exhibit additional cytotoxicity. They can bind to lipids, proteins, deoxyribonucleic acid (DNA), disrupting their normal function, causing cell necrosis (Hauck and Bernlohr, 2016). The process of lipid peroxidation disrupts DNA, protein, and enzyme activity and acts as a molecular activation signaling pathway that initiates cell death (Łuczaj et al., 2017). Ferroptosis, as an iron dependent programmed cell death mode, is closely related to lipid peroxidation. Its essence is the depletion of glutathione (GSH) and the decrease of glutathione peroxidase (GPx) activity, which leads to the inability of lipid oxides to be metabolized through the glutathione peroxidase 4 (GPX4) catalyzed glutathione reductase reaction. Subsequently, divalent iron ions oxidize lipids to produce ROS, thereby promoting the occurrence of ferroptosis (Jiang et al., 2021).

### 3.2 The hazards of lipid peroxides

A rising body of research in the last several years has shown that elevated lipid peroxidation is a significant risk factor for the onset of pulmonary diseases. A redox status imbalance and elevated lung lipid peroxidation products are commonly linked to the etiology of idiopathic pulmonary fibrosis (Tsubouchi et al., 2019). Long-chain acyl-CoA synthetase 4 (ACSL4) gene deletion inhibits lipid peroxidation by lowering PUFA-containing membrane phospholipids on cell membranes, thereby preventing pulmonary toxicity and chemically induced lung injury (Tomitsuka et al., 2023). Using a hypoxic pulmonary artery smooth muscle cell (PASMC) model and a hypoxic mouse model, researcher clarified the role of G protein-coupled receptor 146 (GPR146) in the regulation of lipid peroxidation in pulmonary hypertension. Specifically, hypoxia causes a significant amount of ROS to be released, upregulates GPR146 expression in PASMCs, and induces the expression of 5-lipoxygenase. This process increases the lipid peroxidation product MDA and encourages PASMC proliferation and pulmonary vascular remodeling, aggravating pulmonary hypertension (Huang et al., 2023). Lipid peroxidation caused by severe oxidative stress may contribute to tumor progression by inducing oxidative damage of genetic material, lipids, and proteins, regulating signaling molecules, affecting cell growth and chronic inflammation (Prasad and Srivastava, 2020). However, new research suggests that tumor progression linked to increased lipid peroxidation may actually cause the tumor to decay through necrosis or even apoptosis (Živković et al., 2017).

**FIGURE 2:**
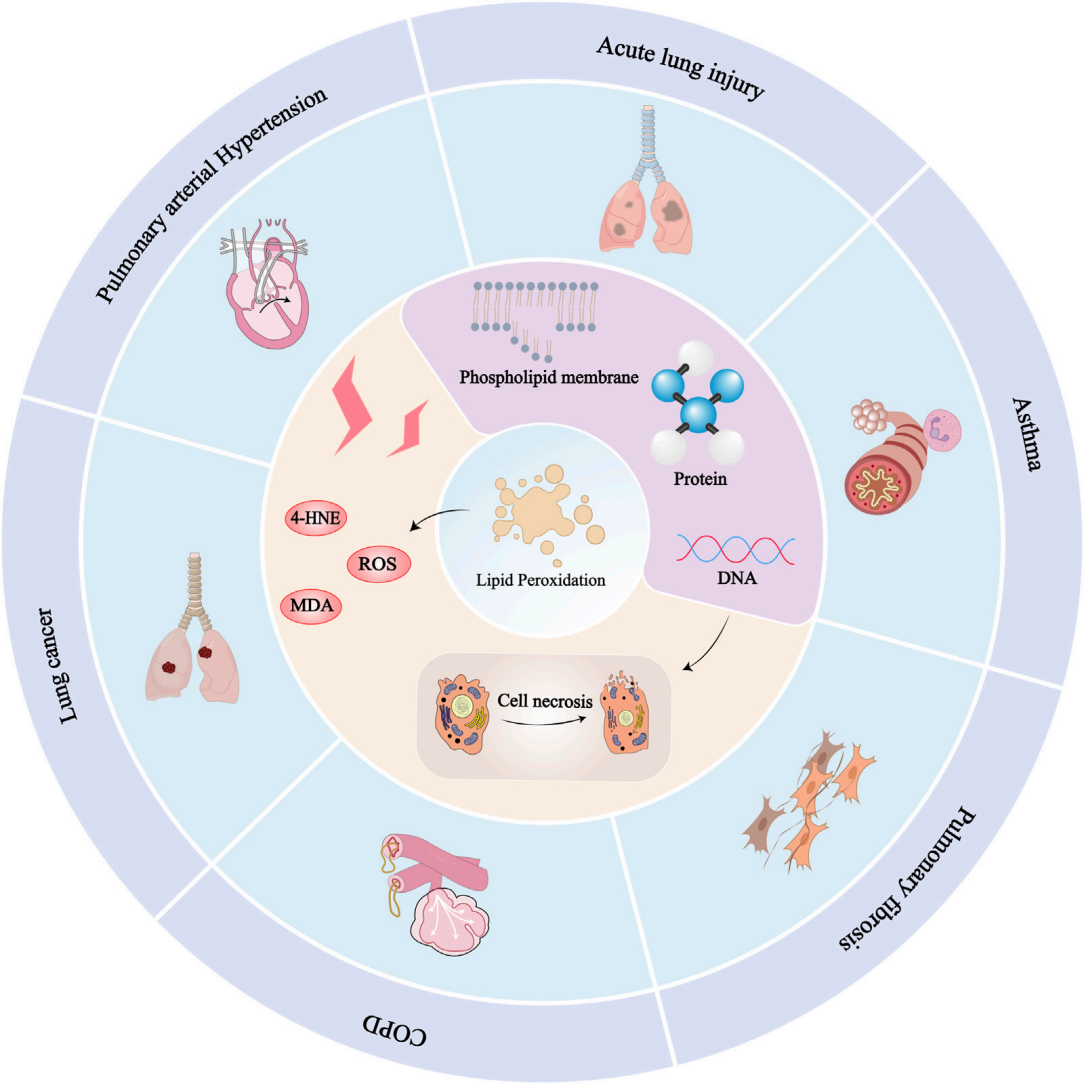
Relationship between lipid peroxidation and various lung diseases: Since lipids are responsible for maintaining the integrity of cell membranes, extensive peroxidation of lipids alters the assembly, composition, structure, and dynamics of lipid membranes. In addition to highly reactive compounds, lipid peroxidation can further generate ROS or degrade into reactive compounds that can cross-link DNA and proteins, causing cellular, tissue, and organ damage and ultimately impairing the body’s physiological mechanisms. Lung diseases such as ALI, asthma, pulmonary fibrosis, pulmonary hypertension, COPD, and lung cancer can be caused by lipid peroxidation.

In China, people have been using traditional Chinese medicine to treat respiratory system diseases for thousands of years. In modern times, various active monomers (such as flavonoids, alkaloids, terpenoids) have been extracted from Chinese herbal medicine and have been proven to have various biological activities such as antioxidant and anti-inflammatory properties (Gu et al., 2014; Liu, 2019). Especially some studies have found that natural ingredients can limit lipid peroxidation in the treatment of lung diseases through multiple pathways and targets. The main mechanism involved in this process is to regulate several critical signaling pathways such as the cystine/glutamate antiporter (System X_c_^−^)/GSH/GPX4 axis, ROS pathway, and nuclear factor erythroid 2-related factor 2 (Nrf2) pathway. Figure 2 shows the relationship between lipid peroxidation and various lung diseases.

#### 3.2.1 System X_c_^−^/GSH/GPX4 axis

The System X_c_^−^/GSH/GPX4 axis is one of the keys to the regulation of lipid peroxidation. Among them, System X_c_^−^ is the key upstream node, GSH is the main cofactor, and GPX4 is the central regulator (Li et al., 2022). System X_c_^−^ is a membrane-bound amino acid antiporter composed of Solute Carrier Family 7 Member 11 (SLC7A11) and Solute Carrier Family 3 Member 2 (SLC3A2). GSH synthesis depends on System X_c_^−^ which inputs cysteine into cells to exchange intracellular glutamate (Bridges et al., 2012). GPx clears intracellular peroxides by catalyzing the reaction between GSH and peroxides. It can convert hydroperoxides into non-toxic lipid alcohols, thereby inhibiting lipid peroxidation and protecting cells from oxidative damage (Zhang et al., 2024). In the GPx catalytic reaction, a disulfide bond is formed between two GSH molecules to form glutathione disulfide. Glutathione reductase (GR) recycles glutathione disulfide to GSH while oxidizing β-nicotinamide adenine dinucleotide phosphate (β - NADPH2), which maintains the reduced state of GSH and ensures its normal antioxidant function (Lu, 2013).

Therefore, GPX4 plays an important role in preventing lipid peroxidation by using GSH as an essential cofactor. The decrease in GSH levels can directly affect GPX4 activity, increase intracellular lipid peroxidation, and ultimately promote ferroptosis, inducing a series of diseases (Yang et al., 2014; Seibt et al., 2019).

#### 3.2.2 Scavenging ROS

ROS is a general term for a large class of oxidants, including superoxide anion radicals (O_2_^·−^), hydroxyl radicals (·OH), and non-radical oxidants such as H_2_O_2_ (Zorov et al., 2014). The main source of ROS is mitochondrial respiratory chain and NADPH oxidase (Brown and Griendling, 2015; Sarniak et al., 2016). ROS is a key component in mediating lipid peroxidation (Wang et al., 2023a). Chemical, physical, and biological factors can individually or synergistically disrupt the balance of redox reactions in lung tissue, leading to an increase in ROS levels produced in the airways. This reaction is manifested in the body as an increase in oxidative stress markers in sputum, respiration, lungs, and blood. ROS, whether directly or through the formation of lipid peroxidation products such as 4-hydroxy-2-nonanal, may play a role in enhancing inflammation by activating stress kinases (JNK, MAPK, p38) and redox-sensitive transcription factors such as NF-κB and AP-1 (Rahman and MacNee, 2000). At the same time, the increase of ROS will also lead to the increase of the expression of Bax, caspase-3, caspase-7 and caspase-9, and the decrease of the expression of Bcl-2, thereby directly activating apoptosis (Zhang et al., 2023).

#### 3.2.3 Nrf2 pathway

Nrf2 is expressed in all cell types and is the main transcription factor regulating antioxidant enzyme expression mediated by antioxidant response elements, but its baseline protein level is usually low in a steady-state environment. Under basic conditions, Nrf2 is isolated in the cytoplasm by actin binding protein Keap1, which can bind to Nrf2 and target it for ubiquitination dependent proteasome disruption (Nguyen et al., 2009). When the lungs are stimulated by various factors and experience oxidative stress, Nrf2 dissociates from Keap1, translocates to the nucleus, binds to antioxidant response elements, and activates antioxidant genes (Zhao et al., 2017). Nrf2 can induce the expression of multiple cell protective genes, for example, heme oxygenase-1 (HO-1), as an antioxidant and anti-inflammatory enzyme, is mainly regulated by Nrf2 activation and can play a protective role against oxidative stress (Paine et al., 2010). Multiple studies have shown that this process has pathophysiological effects on lung diseases such as ALI (Xu et al., 2021) and allergic airway asthma (Jiang et al., 2024). In addition, Nrf2 can indirectly regulate the expression of other antioxidant enzymes such as superoxide dismutase (SOD), GSH, GPx, and catalase (CAT). These enzymes regulate the redox homeostasis in the body by reducing lipid peroxides, allowing the body to recover from oxidative stress to a normal physiological state (McMahon et al., 2006; Dodson et al., 2019; Chen et al., 2024a). Other studies have shown that Nrf2-mediated inhibition of pro-inflammatory cytokine gene induction is not affected by ROS level, and its role in inflammatory inhibition needs to be further explored (Kobayashi et al., 2016). In addition, due to the regulatory effect of Nrf2 on interferon, it is generally believed that it also plays a special role in antiviral immunity (Herengt et al., 2021). Therefore, the Nrf2 pathway has become an important therapeutic molecular target, and activation of the Nrf2 pathway may be a promising way to ameliorate lipid peroxidation and inhibit inflammation in pulmonary diseases.

## 4 Multi-mechanism regulation of lipid peroxidation by natural ingredients in lung diseases

**FIGURE 3:**
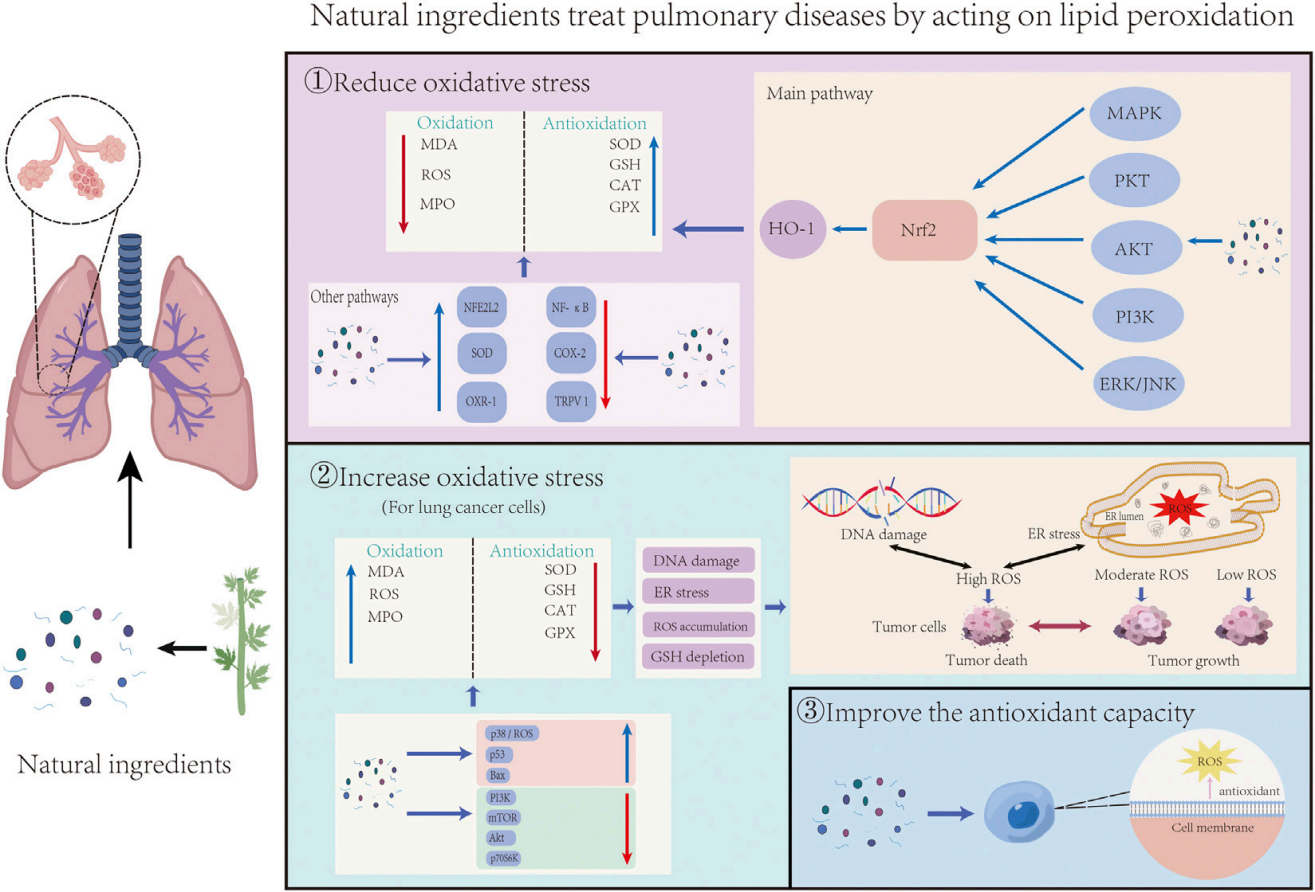
The general process of natural ingredients acting on lung diseases by influencing lipid peroxidation was demonstrated. It can be divided into three parts: Natural ingredients reduce oxidative stress and inhibit lipid peroxidation through Nrf2 and other pathways; For tumor cells, natural ingredients promote lipid peroxidation and apoptosis by increasing the level of oxidative stress; Natural ingredients prevent lipid peroxidation-related diseases by increasing the antioxidant capacity of cells.

In recent years, many researchers have studied natural ingredients’ pharmacological effects and potential molecular mechanisms in treating lung diseases through lipid peroxidation. Abnormal lipid peroxidation can activate ferroptosis, leading to the development and progression of ALI, asthma, pulmonary fibrosis, pulmonary arterial hypertension, COPD, and other respiratory diseases. However, lipid peroxidation also helps to inhibit the growth and spread of tumor cells in lung cancer. Resistance to ferroptosis may promote tumorigenesis and mediate tumor cell resistance to therapeutic drugs. Table 1 provides a summary of natural ingredients used in the treatment of pulmonary diseases, highlighting their compound types, experimental models, relevant test dose ranges, and mechanisms of lipid peroxidation regulation. Figure 3 illustrates the general process by which natural ingredients affect lipid peroxidation in lung diseases.

**TABLE 1:** Natural ingredients regulating lipid peroxidation in pulmonary disease.

Disease	Compound	Classification	Cell/animal model	Dose/Concentration	Control group	Mechanisms	Ref
Acute lung injury	Myricetin	Flavonoids	Male C57BL/6 mice induced by CLP	100 mg/kg, i.g	-	Restore the activity of antioxidant enzymes; Enhancing the Nrf2/HO-1 signaling	Xu et al. (2021)
	Astragalin	Flavonoids	BEAS-2B cells induced by LPS or PBS Male SD rats induced by LPS (10 mg/mL)	0–200 μM 50 mg/kg, p.o		Activate the Nrf2/HO-1 pathway	Zheng et al. (2019)
	Chrysin	Flavonoids	Rat pups induced by hyperoxia (90%–95% O_2_)	(20 mg/kg/day, i.p) for 10 days	-	Restore antioxidant levels	Ozdemir et al. (2021)
	Rutin	Flavonoids	Adult male ICR mice induced by LPS (100 g/50 μL)	0, 1, 10, 100 mol/kg, i.p	Dexamethasone (1 mg/kg, i.p); Desferrioxamine (20 mg/kg, i.p)	Enhance the activity of antioxidant enzymes; Increase HO-1 expression	Yeh et al. (2014)
	Acetovanillone	Phenols	Male Wistar rats induced by CP (200 mg/kg, i.p)	(100 mg/kg, p.o) for 10 days	-	Reduce ROS; Regulate Keap1-Nrf2/HO-1 pathway; Activate the PI3K/Akt/mTOR signaling pathway	Abd El-Ghafar et al. (2021)
	Colchicine	Alkaloids	Male SD rats induced by 5% sodium taurocholate	(0.5 mg/kg/day, i.g) for 7 days	-	Increase GSH content and activity of SOD; Restore Nrf2 and HO-1 expression	Zhang et al. (2022)
	Crocin	Terpenoids	Male SD rats induced by CS	(50 mg/kg/day, TIW, i.p) for 2 months	-	Increase GSH content and activities of SOD, CAT and GPX; Activate Nrf2 pathway	Dianat et al. (2018)
	Zerumbone	Terpenoids	Male ICR mice induced by LPS (100 μg/50 μL, IT)	0, 0.1, 1, 10 μmol/kg, i.p	Dexamethasone (1 mg/kg, i.p)	Enhance the activity of antioxidant enzymes; Upregulate of Nrf2/HO-1 pathway	Leung et al. (2017)
	Thymoquinone	Quinones	Male Swiss albino rats induced by B(a)P (50 mg/kg, p.o)	(50 mg/kg, TIW, i.p) for 8 weeks	-	Enhance the activity of antioxidant enzymes	Alzohairy et al. (2021)
	Panaxydol	Saponins	BEAS-2B cells induced by LPS (10 μg/mL)	10, 20, 40 μg/mL	-	Upregulate Keap1-Nrf2/HO-1 pathway	Li et al. (2021)
Asthma	Fisetin	Flavonoids	BEAS-2B cells induced by TNF-α (10 ng/mL) Female BALB/c mice induced by OVA (50 μg)	0–30 μM 5, 10 mg/kg, i.p	-	Reduce ROS production; Activate the Nrf2/HO-1 pathway; Raise the level of GSH	Wu et al. (2022)
	Tectorigenin	Flavonoids	Male BALB/c mice induced by OVA	(10, 25 mg/kg, p.o) for 14 days	Dexamethasone (1.5 mg/kg, p.o.)	Increase the level of antioxidant; Activate Keap1/Nrf2/HO-1 pathway	Jiang et al. (2024)
	Tectochrysin	Flavonoids	C57BL/6 mice induced by ST (20 μg)	(2.5, 5 mg/kg, i.p) for 7 days	Dexamethasone (2 mg/kg, i.p)	Enhance the activity of CAT and GPX	Fang et al. (2021)
	Sophoraflavanone G	Flavonoids	BEAS-2B cells induced by TNF-α (10 ng/mL) Female BALB/c mice induced by OVA	0–30 μM 5, 10 mg/kg, i.p	-	Increase the expression of SOD, CAT, and GSH	Wang et al. (2022b)
	Licochalcone A	Flavonoids	BEAS-2B cells induced by TNF-α and IL-4 (10 ng/mL) Female BALB/c mice induced by OVA	0–20 μM 5 mg/kg, i.p	-	Reduce the expression of COX-2 and ROS; Upregulate Nrf2/HO-1 pathway	Huang et al. (2019)
	Esculentoside A	Glycosides	A549 cells Female BALB/c mice induced by OVA	0, 5, 10, 20 mg/l 15 mg/kg/day, i.p	Dexamethasone (2 mg/kg, i.p)	Upregulate Nrf2/HO-1 pathway; Improve the levels of antioxidants	Ci et al. (2015)
Pulmonary fibrosis	Rutin	Flavonoids	Male SD rats induced by BLM (2 mg/kg)	(50, 100 mg/kg/day, i.g) for 3 weeks	Dexamethasone (0.5 mg/kg/day, i.g) for 3 weeks	Restore the activity of GSH and SOD	Bai et al. (2020)
	Hyperoside	Flavonoids	Male C57BL/6 mice induced by BLM (2 mg/kg)	(50 mg/kg/day, i.p) for 14 days		Enhance the activity of SOD	Huang et al. (2020)
	Thymoquinone	Quinones	Male NMRI mice induced by PQ (20 mg/kg, i.p)	(20, 40 mg/kg) for 14 or 28 days	-	Restore the activity of SOD and CAT	Pourgholamhossein et al. (2016)
	Zingerone	Phenols	Male Wistar-albino rats induced by BLM (5 mg/kg)	(50, 100 mg/kg, p.o) for 14 days	-	Enhance the activity of SOD and GPX	Gungor et al. (2020)
	Dihydroquercetin	Flavonoids	HBE cells induced by SiO_2_ (50 ug/mL) Male C57BL/6 mice induced by SiO_2_ suspension for 1 week	40 μM; (10, 50 mg/kg, bid, p.o) for 14 days	-	Clear ROS; Raise the level of GSH and GPX; Activate Nrf2 pathway	Yuan et al. (2022)
	Fraxetin	Coumarins	MLE-12 cells induced by BLM (0.5 nM) C57BL/6 mice induced by BLM (1.4 U/kg)	10, 20, 40 μM 10 mg/kg, qod, i.p	-	Clear ROS; Restore the level of GSH and GPX	Zhai et al. (2023)
Pulmonary arterial hypertension	Cyanidin-3-O-β-glucoside	Flavonoids	Male SD rats induced by MCT (60 mg/kg)	(200/400 mg/kg, i.g) for 28 days	-	Enhance the activity of SOD	Ouyang et al. (2021)
	Baicalein	Flavonoids	Male SD rats induced by MCT (60 mg/kg)	(50/100 mg/kg/d, p.o) for 28 days	-	Enhance the activity of SOD	Shi et al. (2018)
	Diosgenin	Steroids	Male Wistar rats induced by MCT (60 mg/kg)	(100 mg/kg/d, p.o) for 21 days	-	Restore GSH and SOD; Inhibit AKT and ERK pathways	Parama et al. (2020)
	Resveratrol	Phenols	SD rats induced by hypoxia for 28 days	(40 mg/kg/d, i.g) for 28 days	-	Restore GSH and SOD; Inhibit AKT and ERK pathways	Xu et al. (2016)
	Arctigenin	Terpenoids	MCT (60 mg/kg) treated SD rats	(50 mg/kg/d, i.p) for 28 days	-	Restore the activity of SOD	Jiang et al. (2018)
	18β-Glycyrrhetinic Acid	Terpenoids	Male SD rats induced by MCT (60 mg/kg)	(100, 50, 25 mg/kg/d, p.o) for 21 days	-	Restore the activity of SOD	Zhang et al. (2019)
	Free and nanoemulsified β-caryophyllene	Terpenoids	Male Wistar rats induced by MCT (60 mg/kg)	(176 mg/kg/d, i.g) for 14 days	-	Enhance the activity of antioxidant enzymes; Decrease the expression of endothelin-1 receptors	Carraro et al. (2024)
	Crocin	Terpenoids	Male SD rats induced by MCT (60 mg/kg)	(7.5, 15, 30 mg/kg/d, i.p) for 21 days	-	Enhance the levels of SOD and GSH	Dianat et al. (2020)
	Berberine	Alkaloids	Male SD rats injected with MCT (60 mg/kg)	(10, 20, 30, 40 mg/kg/d, i.p) for 3 weeks	-	Enhance the activity of SOD, GPx, CAT	Beik et al. (2023)
	Carvacrol	Phenols	PASMC cells (incubated with a gas mixture containing 3% O_2_, 5% CO_2_, and 92% N_2_ for 24 h) Adult male Wistar rats randomized to 9 days of hypoxic environments (FiO2 = 0.12)	600 μM, 24h; (25, 50, 100 mg/kg/d, i.p) for 9 days	-	Enhance the activity of SOD and GSH	Zhang et al. (2016)
COPD	Luteolin	Flavonoids	A549 Cells induced by CSE Male C57BL/6J mice induced by CS + LPS exposure	15, 30 μM, 12 h (50, 100 mg/kg/d, i.g) for 15 weeks	-	Enhance the activity of SOD and GSH	Zhou et al. (2023)
	Hesperidin	Flavonoids	C57BL/6 mice injected with 100% cigarette smoke extract (0.3 mL)	(25, 50 mg/kg/d, i.p) for 21 days	Budesonide (2 mg/kg)	Enhance the activity of SOD and CAT	Wang et al. (2020)
	Gallic acid	Phenols	Male Balb/c mice induced by ET + LPS	(200 mg/kg/d, i.p) for 28 days	-	Restore the activity of SOD	Singla et al. (2021)
	Betulin	Terpenoids	Male ICR mice induced by CSE for 8 weeks	(20, 40 mg/kg/d, i.g) for 8 weeks	Dexamethasone (2 mg/kg)	Enhance the activity of SOD and CAT	Chunhua et al. (2017)
Lung cancer	Luteolin	Flavonoids	The human NSCLC cell lines, NCI-H1299 and -H460 BALB/cAnNCrj-nu/nu strain mice	10, 20, 30, 40, 50, 100 μM, 72 h (10 mg/kg/d, s.c) for 35 days	-	Inhibit p38 MAPK	Cho et al. (2015)
	Formosanin C	Steroids	The NCI-H1299(CRL-5803), NCI-H1975(CRL-5908), A549(CRM-CCL-185),293T (CRL-3216) cells Male C57BL6/J mice inoculated with 5 × 105 LLC-1 cells after 1 week of acclimation	0–2 μM, 24 h/48 h; (0.5/1 mg/kg, i.p, qod) for 16 days	Cisplatin (1 mg/kg)	Influence myo-inositol and related pyruvate metabolism, glycolysis/gluconeogenesis, and citrate cycle; glutathione metabolism	Li et al. (2023)
	Sinapine	Alkaloids	Human NSCLC cell lines (A549, SK, H661, H460, H460 p53−/−, A549 p53−/−) BALB/c mice (tumour cells were implanted (1x10^7^ cells/mL) into the left frontal axils)	2, 5, 10, 20 μM; (10, 20, 40 mg/kg/d, i.v) for 30 days	-	Intensifier transferrin/transferrin receptor expression	Shao et al. (2022)
	Dihydroartemisinin	Terpenoids	16HBE cells, Lewis mouse lung cancer cell line Female C57BL mice	30/60 μg/mL, 24 h; (5/10 mg/kg/d, i.p) for 3 days	*In vitro*: Doxorubicin (5 μM) *In vivo*: Doxorubicin (5 mg/kg b.w.)	Reduce GPX4; Intensify expression of COX-2; Enhance ferroptosis	Han et al. (2023)
	Resveratrol	Phenols	Human lung adenocarcinoma A549 cell line	60 μM resveratrol and/or 10 μM As_2_O_3_ for 24 h	-	Reduce the SOD activity; Imbalance in the chemical-antioxidant system	Gu et al. (2015)
	Purpurin	Quinones	Human lung adenocarcinoma A549 cell line, human dermal fibroblasts	30 μM, 24 h/48 h	-	Enhance lipid peroxidation mediated by ROS	Bo et al. (2021)

MDCK, Madin-Darby Canine Kidney; SD, sprague dawley; PBS, Phosphate-Buffered Saline; ICR, institute of cancer research; CSE, cigarette smoke exposure; MCT, monocrotaline; CP, cyclophosphamide; PAEC, Pulmonary Artery Endothelial Cell; PASMC, pulmonary artery smooth muscle cell; LPS, lipopolysaccharide; ET, elastase; NSCLC, Non-Small Cell Lung Cancer. (-: blank control, no medication or treatment).

### 4.1 Acute lung injury

ALI and its more serious form, acute respiratory distress syndrome (ARDS), as respiratory diseases with high mortality rates, are manifested by acute hypoxemic respiratory failure, increased alveolar permeability and severe alveolar edema with normal cardiac filling pressures (Shaw et al., 2019). The clinical therapies applied in ALI/ARDS mainly include pulmonary protective ventilation and limited fluid management supplemented by glucocorticoids, inhaled pulmonary vasodilators, neuromuscular block, and extracorporeal membrane oxygenation (Liu et al., 2022).

Many studies have found that in mouse models with ALI/ARDS, an increase in lipid peroxidation marker MDA levels and a significant decrease in antioxidant enzyme activities such as SOD, CAT, and GPx have been observed (Yeh et al., 2014; Dianat et al., 2018; Su et al., 2019). An increase in MDA levels indicates an increase in lipid peroxidation levels, which can disrupt the integrity of phospholipid membrane function, cause the release of large amounts of H_2_O_2_, and toxicity to cells, ultimately resulting in corresponding tissue injury (Gaschler and Stockwell, 2017). In addition, the increase in lipid peroxidation levels can also induce ferroptosis, leading to the occurrence of inflammation. Inflammatory response is an important factor in lung tissue injury. Results of an *in vitro* study showed that ferroptosis mediated inflammation in LPS (lipopolysaccharide)-treated BEAS-2B cells, and treatment with Panaxydol in the study attenuated LPS-induced inflammation and ferroptosis by up-regulating the Keap1-Nrf2/HO-1 pathway (Li et al., 2021). Keap1-Nrf2/HO-1 signaling is considered one of the important endogenous anti-oxidative stress pathways and a critical target in inflammation-related diseases (Mills et al., 2018; Lu et al., 2019).

The natural ingredients therapy for ALI is mainly achieved by enhancing the antioxidant system and alleviating lipid peroxidation caused by oxidative stress, with the Nrf2/HO-1 pathway being its core mechanism.

Myricetin is a flavonoid compound widely found in many natural plants including bayberry, and was originally isolated from the bark of the tree Myrica rubra (*Myrica rubra* (Lour.) Siebold & Zucc.) (Song et al., 2021). Myricetin has multiple biological characteristics, including anti-inflammatory (Hou et al., 2018), antitumor (Stoll et al., 2019), antibacterial (Jiang et al., 2019), and anti-obesity effects (Hu et al., 2018), especially against oxidative stress. It has been shown that myricetin can target lipid peroxidation to treat cecal ligation and puncture-induced ALI by decreasing MDA and 4-HNE levels and up-regulating SOD, CAT, and GPx activities. After establishing a mouse sepsis model with Nrf2 knockout and a control group, it was found that the knockdown of Nrf2 showed an inhibitory effect on myricetin treatment for sepsis. This also indicates that myricetin exerts protective effects on sepsis-associated lung injury dependent on Nrf2. In addition, myricetin ameliorated lung mitochondrial dysfunction and inhibited ROS production in septic mice (Xu et al., 2021).

Astragalin (kaempferol 3-glucoside) is one of the naturally occurring flavonoids, which can effectively inhibit lipid peroxidation and treat pulmonary diseases (Riaz et al., 2018). *In vitro* experiments, astragalin induced Nrf2 activation and HO-1 upregulation in BEAS-2B cells. When Nrf2 was silenced by siRNA, the effect of astragalin on HO-1 upregulation was eliminated. This suggests that astragalin also upregulates HO-1 in an Nrf2-dependent manner. In addition, compared with the LPS treatment group, astragalin treatment significantly reduced the levels of TNF-α and MMP-9, inhibited LPS-induced lung histological changes, including edema and inflammatory cell infiltration, which may be related to the Nrf2/HO-1 pathway (Zheng et al., 2019).

The levels of endogenous antioxidants such as SOD, GSH and GPx decreased significantly in rats with hyperoxia-induced lung injury, and these changes returned to normal after chrysin treatment, suggesting that chrysin can play a defensive role against lipid peroxidation by enhancing the antioxidant defense system. In addition, chrysin as a flavonoid itself has potential antioxidant effects and can mitigate lipid peroxidation (Naz et al., 2019; Ozdemir et al., 2021).

Rutin is a natural polyphenolic flavonoid found in fruits and vegetables. In a positive control group of a study, dexamethasone pretreatment reduced LPS-induced histopathological damage. Rutin also attenuated LPS-induced pathological damage in mouse lung tissue in a concentration-dependent manner, including bleeding, interstitial edema, thickening of alveolar walls, and infiltration of polymorphonuclear granulocytes into lung parenchyma and alveolar spaces. This protective effect is related to the inhibition of lipid peroxidation. Rutin can increase the activity of antioxidant enzymes (SOD, CAT, and GPx) and increase the expression of HO-1. In addition, other potential mechanisms involved include reducing the secretion of pro-inflammatory cytokines such as TNF, IL-1, and IL-6, and inhibiting MAPK phosphorylation. In another positive control group, desferrioxamine not only reduced LPS-induced polymorphonuclear granulocytes infiltration, but also restored SOD and GPx activity. Rutin has been shown to improve LPS-induced ALI more effectively than desferrioxamine. These experimental results support the potential use of rutin as a therapeutic agent to prevent ALI associated with direct infection by Gram-negative bacteria (Yeh et al., 2014).

There is also a potent NADPH oxidase inhibitor, acetovanillone, commonly known as apocynin. In the ALI rat model induced by cyclophosphamide, GSH and antioxidant enzyme activities are reduced, while lung lipid peroxidation and NADPH oxidase activity are increased. After 10 days of oral administration, acetovanillone reduced ROS generation by inhibiting the activity and expression of NADPH oxidase. On the other hand, cytoglobin is an intracellular respiratory globulin that can clear excess ROS and maintain physiological ROS levels (Mathai et al., 2020; Zweier et al., 2021). Acetovanillone can increase the expression of cytoglobin in lung tissue of cyclophosphamide induced ALI rats, which may be another mechanism by which acetovanillone alleviates lipid peroxidation and ALI. More importantly, acetovanillone can activate the Nrf2 pathway, increase the expression of downstream HO-1 and glutamate cysteine ligase catalytic (GCLc), thereby enhancing antioxidant capacity. *In vitro* studies have shown that the combination of cyclophosphamide and acetovanillone is more effective in inhibiting cancer cell growth compared to treatment alone, demonstrating the synergistic effect between the two drugs. This indicates that acetovanillone does not hinder the anti-tumor activity of cyclophosphamide and is a promising drug that can be used to prevent lung injury in chemotherapy patients without affecting the efficacy of chemotherapy drugs (Abd El-Ghafar et al., 2021).

Colchicine is a tricyclic, lipid-soluble alkaloid derived from the plant of the Colchicum autumnale (*Colchicum autumnale* L.) (Angelidis et al., 2018). In severe acute pancreatitis-associated ALI rat plasma, colchicine can inhibit lipid peroxidation in rats by restoring Nrf2/HO-1 signaling, while reducing the expression of ROS and 4-HNE, helping to restore redox homeostasis and protect tissue cells from oxidative stress-induced apoptosis. The experimental results also confirmed that colchicine treatment reduced caspase-3 cleavage and Bax expression in severe acute pancreatitis-associated ALI rats, but increased Bcl-2 expression significantly alleviated cell apoptosis. In addition, the treatment with colchicine also weakened the activation of NF-κB, STAT3, and AKT signals during ALI in rats, which may be related to the ability of colchicine to directly or indirectly clear antioxidants (Zhang et al., 2022).

Zerumbone is a monocyclic sesquiterpene and the major active phytochemical compound extracted from rhizome of Zingiber zerumbet (*Zingiber zerumbet* (L.) Roscoe ex Sm.) (Duñg et al., 1993). Research has shown that zerumbone can reduce LPS-induced ALI by increasing antioxidant enzyme activity (such as SOD, CAT, and GPx) and upregulating the Nrf2/HO-1 pathway. In addition, lung specimens treated with LPS showed significant pathological changes, including neutrophils infiltration, increased alveolar wall thickness, hemorrhage, and hyaline membrane formation. In the positive control group, the glucocorticoid dexamethasone reduced these pathological changes, while zerumbone pre-treatment improved lung lesions in a concentration dependent manner. This suggests the possibility of using zerumbone as an alternative protective agent for ALI directly associated with Gram-negative bacterial infections (Leung et al., 2017).

Crocin is a water-soluble carotenoid, it is also the most important active constituent of saffron (*Crocus sativus* L.). A model of cigarette smoke-induced lung injury in rats given by intraperitoneal injection crocin. According to the experimental results, crocin treatment reduced MDA levels in lung tissues, indicating a significant reduction in free radical-induced lipid peroxidation. In addition, Nrf2 is a crucial regulator of cells against oxidative stress. The experimental results showed that crocin could stimulate Nrf2 by up-regulating the expression of PKC, PI3K and MAPK mRNA, thus up-regulating the Nrf2-GCLc-GSH pathway. Meanwhile, crocin also greatly increased its downstream antioxidant enzyme activities, such as SOD, CAT and GPX, to minimize lung damage caused by exposure to cigarette smoke (Dianat et al., 2018).

Thymoquinone is the main active ingredient of Nigella sativa (*Nigella sativa* L.). Antioxidants slow autoxidation by scavenging substances that trigger peroxidation to produce ROS. The balance between controlled ROS formation and endogenous antioxidant defense is important in inhibiting pathogenesis. The experimental results showed that the oral administration of benzo (a) pyrene to rats in the treated group resulted in a significant increase in MDA concentration and a significant decrease in the levels of several antioxidant enzyme markers, leading to an imbalance in the antioxidant system. Whereas, thymoquinone treatment reversed these changes and increased antioxidant enzyme activities (SOD, CAT). In addition, thymoquinone decreased the expression of pro-inflammatory markers (NF-κB, IL-6, and COX-2), showing antioxidant and anti-inflammatory effects (Alzohairy et al., 2021).

### 4.2 Asthma

Asthma, one of the most common chronic, non-communicable diseases in children and adults, is characterized by variable respiratory symptoms and variable airflow limitation (Papi et al., 2018). The pathogenesis of asthma is often related to factors such as viral respiratory infections, allergy and defective anti-viral immunity, bacterial infections, and allergen exposure (Castillo et al., 2017). Anti-inflammatory and bronchodilator treatments are the mainstay of asthma therapy and are used in a stepwise approach (Papi et al., 2018). Natural ingredients have significant antioxidant activity and can regulate lipid peroxidation levels to treat asthma and alleviate airway hyperresponsiveness. It is generally achieved by weakening the production of ROS, enhancing the activities of GPx, GR, SOD, and CAT, and reducing toxic metabolites such as MDA. In addition, *in vitro* studies have found that IL-6 promotes ferroptosis in bronchial epithelial cells by inducing ROS dependent lipid peroxidation (Han et al., 2021). Therefore, targeting lipid peroxidation can prevent the occurrence of ferroptosis and have a positive effect on asthma. The regulation of lipid peroxidation by natural ingredients in the treatment of asthma is mainly achieved by enhancing the antioxidant system and reducing inflammation of tracheal epithelial cells. During this process, it may also involve pathways such as PI3K/AKT, ERK/JNK, Nrf2.

A common flavonoid found in a wide variety of fruits and vegetables is called fisetin. *In vitro* experiments, BEAS-2B cells treated with fisetin were stimulated with TNF-α/IL-4. The results showed that TNF-α stimulation significantly promoted ROS expression in BEAS-2B cells, while fisetin effectively reduced the expression of pro-inflammatory cytokines (CCL5, MCP-1, IL-8, and IL-6) in BEAS-2B cells, and effectively improved oxidative stress by activating the Nrf2/HO-1 pathway. In addition, fisetin inhibited the activation of the NF-κB signaling pathway in BEAS-2B cells stimulated by TNF-α, reducing phosphorylation of IκB-α and nuclear translocation of p65. Fisetin also inhibited the activation of the MAPK signaling pathway in BEAS-2B cells stimulated by TNF-α, including phosphorylation of p38, JNK, and ERK1/2. *In vivo* studies have shown that fisetin improves lung health in asthmatic mice by increasing GSH levels, inhibiting COX-2 expression, and reducing MDA levels, ultimately reducing lipid peroxidation in lung cells (Wu et al., 2022).

A well-known natural flavonoid aglycone called tectorigenin exists in numerous plants (Rong et al., 2023). Tectorigenin significantly hindered the elevation of the levels of ROS and MDA in the bronchoalveolar lavage fluid (BALF) of the asthma group. It also increased the levels of antioxidants, such as SOD and CAT, exerting its antioxidant capacity to counteract lipid peroxidation and ameliorate asthma-associated oxidative stress. In addition, tectorigenin can also exert antioxidant and anti-inflammatory effects by activating the Keap1/Nrf2/HO-1 pathway in the ovalbumin (OVA)-induced asthma mouse model to alleviate allergic respiratory diseases, which has great potential to provide additional drug options for allergy-related diseases (Jiang et al., 2024). Tectochrysin is also a type of flavonoid compound that can be isolated from propolis. Results demonstrated that tectochrysin could enhance the activity of CAT and GPx in lung tissue, encourage the breakdown of peroxides, and shield shrimp tropomyosin-induced asthma mice from oxidative damage after tectochrysin was given intraperitoneally to a mouse asthma model for 6 days. In addition, treatment with tectochrysin or dexamethasone significantly reduced the levels of IL-4 and IL-5 as well as the IL-4/IFN-γ ratio in BALF of asthmatic mice, indicating the potential of tectochrysin as a therapeutic agent for asthma (Fang et al., 2021).

Research has found that Sophoraflavanone G from Sophora flavescens (*Sophora flavescens* Aiton) can reduce the production of MDA in the lungs of OVA-sensitized asthmatic mice and increase the expression of SOD, CAT, and GSH. This indicates that Sophoraflavanone G can alleviate lipid peroxidation by regulating the antioxidant system and protect against lung injury in asthmatic mice. In addition, Sophoraflavanone G can regulate the expression of cytokines and chemokines in BALF and lung tissue. In BALF, compared with OVA-induced asthma mice, Sophoraflavanone G significantly reduced the levels of IL-4, IL-5, IL-13, TNF-α, IL-6, CCL11, and CCL24. In asthma patients, Th2 cells secrete more IL-4 to induce B cell activation, leading to excessive IgE secretion (Moran and Pavord, 2020). The study found that the levels of IL-4, IL-5, and IL-13 in spleen cells of asthmatic mice treated with Sophoraflavanone G were significantly reduced, and the levels of OVA-IgG1 and OVA IgE were also significantly reduced as a result (Wang et al., 2022b).

Licochalcone A is a flavonoid compound isolated from Glycyrrhiza uralensis (*Glycyrrhiza uralensis* Fisch. Ex DC.). OVA-sensitized mice were treated with licochalcone A via intraperitoneal injection. The experimental results showed that licochalcone A can reduce the expression of MDA, increase the synthesis of GSH, upregulate the Nrf2/HO-1 pathway, and reduce the expression of COX-2 and intracellular ROS. This suggests that licochalcone A may protect the lungs of asthmatic mice from oxidative stress by regulating the antioxidant system. In addition, compared with OVA-sensitized asthmatic mice, licochalcone A reduced inflammation of tracheal epithelial cells by inhibiting the expression of IL-6, COX-2, CCL11, CCL-24, and MUC5AC. These experimental results indicate that licochalcone A has excellent potential in improving asthma inflammation and oxidative stress (Huang et al., 2019). Esculentoside A is a saponin isolated from the root of Phytolacca esculenta (*Phytolacca esculenta* Van Houtte). Research has found that both Esculentoside A and dexamethasone (positive controls) significantly alleviate asthma reactions, including airway inflammation, eosinophil migration to the lungs, excessive mucus secretion, and a decrease in Th2 cytokines and IgE, while enhancing antioxidant capacity by increasing the levels of SOD, CAT, and GSH. *In vitro* experiments showed that Esculentoside A upregulated the Nrf2/HO-1 pathway in a dose-dependent manner after treating A549 cells with Esculentoside A (20 mg/L). Further research has found that after inhibiting Nrf2 with Nrf2 siRNA, the regulatory effects of Esculentoside A on inflammation and oxidative stress are canceled, indicating that the effect of Esculentoside A depends on the activation of the Nrf2 signaling pathway. Esculentoside A also increased the mRNA expression of antioxidant enzymes, such as HO-1 and glutathione S-transferase, through the Nrf2 signaling pathway. These results confirm that Esculentoside A inhibits the development of lipid peroxidation by enhancing the antioxidant system and is a potential new drug with anti-inflammatory and antioxidant properties (Ci et al., 2015).

### 4.3 Pulmonary fibrosis

Pulmonary fibrosis involves a spectrum of chronic and often progressive lung diseases that primarily affect the interstitium in the lungs. A combination of inflammation and fibrosis in the interstitial space leads to impaired gas exchange resulting in dyspnea (Kim et al., 2015; Raghu et al., 2022), impaired quality of life, and in many patients eventually respiratory failure and death. At present, pulmonary fibrosis is still incurable. Lung transplantation is a feasible treatment option, but it still has limitations, such as not being suitable for elderly populations with multiple coexisting diseases (Somogyi et al., 2019). Our focus is on slowing the progression of pulmonary fibrosis and finding effective therapeutic targets. According to reports, H_2_O_2_, MDA levels, and aldehyde 4-HNE are elevated in lung fibroblasts from patients with pulmonary fibrosis. This is related to the reduction of enzymes such as GPX4 in pulmonary fibrosis fibroblasts, which also contributes to the differentiation of myofibroblasts during the fibrosis process (Tsubouchi et al., 2019). On the other hand, peroxidation products have also been shown to induce the expression of pro-fibrotic molecules such as TGF-β and fibronectin (Tsukagoshi et al., 2002). TGF-β1 is one of the most common cytokines that cause fibrosis and plays a critical role in the development of the extracellular matrix. These mechanisms include: activating Smad-dependent and non-dependent signaling pathways, promoting transcription of collagen genes; Regulating the expression of microRNAs (such as miR-29, miR-326) to stabilize the translation and secretion of collagen; As one of the main inducers of epithelial-mesenchymal transition (EMT), it activates mesenchymal genes and inhibits epithelial gene expression through Smad dependent pathways; Induce the expression of EMT transcription factors such as Snail1 and Twist (Kim et al., 2018).

In the experimental models of pulmonary fibrosis induced by BLM (bleomycin), cigarette smoke, and paraquat, it was observed that natural ingredients could reduce lipid peroxidation toxic products (such as MDA, 4-HNE), enhance antioxidant enzyme activities such as SOD, GPx, CAT, regulate the Nrf2 pathway, reduce lipid peroxidation induced lung tissue damage. More importantly, natural ingredients can also reduce the expression of pro-fibrotic factors such as TGF-β1, thereby alleviating the progression of pulmonary fibrosis.

In a rat lung fibrosis model induced by BLM, dexamethasone (positive control group) and rutin significantly reduced the protein expression rates of fibrosis biomarkers (a-SMA, collagen I and collagen III) and TGF-β1, improved bleeding, thickening of alveolar septa, infiltration of alveolar wall cells, and necrosis of alveolar tissue. This may be related to the enhanced antioxidant system in rats, as experiments have also observed that rutin can reduce the production of lipid peroxidation toxic products (such as MDA, 4-HNE), enhance the activity of antioxidant enzymes such as SOD, GPx, CAT, regulate the Nrf2 pathway, thereby maintaining the balance of oxidants/antioxidants in rats and reducing lipid peroxide induced lung tissue damage (Bai et al., 2020).

Another type of flavonoid compound hyperoside, which is extracted from Rhododendron brachycarpum (*Rhododendron brachycarpum* D.Don ex G.Don) (Ye et al., 2017). Injecting hyperside intraperitoneally into a BLM-induced pulmonary fibrosis mouse model, it was found that hyperside intervention significantly reduced MDA content and increased SOD activity, indicating that hyperside significantly inhibited oxidative stress and lipid peroxidation. In addition, hyperoside inhibits the induction of EMT by BLM *in vivo*, specifically by reducing the expression of a-SMA, collagen I, and TGF-β1 compared to the BLM group. Hyperoside also inhibits the AKT/GSK3β signaling pathway, which may also have an inhibitory effect on EMT. These findings provide a promising candidate drug for the treatment of pulmonary fibrosis (Huang et al., 2020).

In addition, studies have measured oxidative stress markers in lung tissue of mice stimulated by paraquat, and the results showed that the level of lipid peroxidation in mice significantly increased, while the activities of SOD and CAT enzymes decreased. Administration of thymoquinone reversed these results in a dose-dependent manner. In addition, compared with the paraquat group, the mRNA expression of COL1A1, COL4A1 and α-SMA in mice treated with thymoquinone decreased in a dose-dependent and time-dependent manner, indicating that thymoquinone can inhibit the activation of pro-fibrotic genes and extracellular matrix deposition. Meanwhile, compared with the paraquat group, dose-dependent thymoquinone treatment inhibited the mRNA expression of TGF-β1 (Pourgholamhossein et al., 2016).

In a BLM-induced rat model of pulmonary fibrosis, it was found that BLM disrupts the balance between oxidant/antioxidant defense mechanisms and induces oxidative stress by decreasing the activity of SOD and GPx, which leads to elevated levels of the lipid peroxidation marker MDA, and zingerone treatment reversed these changes. In addition, IL-1β promotes fibrosis development by disrupting alveolar structure and enhancing collagen deposition (Kolb et al., 2001), while zingerone (50 and 100 mg/kg) significantly reduced TNF-α and IL-1β levels in BALF, demonstrating strong anti-inflammatory and anti-fibrotic effects (Gungor et al., 2020).

Dihydroquercetin, also known as paclitaxel, is a typical plant flavonoid found in yew, larch, and cedrus brevifolia bark. Research has found that dihydroquercetin can alleviate SiO_2_-induced inflammation and fibrosis in lung tissue. Compared with the SiO_2_ group, dihydroquercetin reduces the levels of pro-inflammatory cytokines (including IL-1β, TNF-α, and TGF-β) in serum and lung homogenate, and significantly decreases the expression of α-SMA, collagen I, and fibronectin. In addition, compared with the SiO_2_ group, dihydroquercetin treatment significantly reduced iron, ROS, MDA, and 4-HNE levels, but significantly increased GSH and GPX4 levels. More importantly, dihydroquercetin treatment significantly reduced the expression of α-SMA, collagen I, and fibronectin in HBE cells, and this effect was significantly reversed by erastin. These results indicate that the stimulation of ferroptosis impairs the anti-fibrotic effect of dihydroquercetin *in vitro* (Yuan et al., 2022).

Fraxetin is a hydroxycoumarin compound extracted from the natural medicinal plant Fraxinus rhynchophylla (*Fraxinus chinensis subsp*. Rhynchophylla). *In vitro* experiments showed that pretreatment with fraxetin upregulated the expression of SLC7A11, and GPX4 in MLE-12 cells treated with BLM, thereby reducing lipid peroxidation levels. In addition, *in vivo* experiments showed that when treating BLM attacked pulmonary fibrosis mouse models with fraxetin, the mRNA expression of fibronectin, as well as the protein level of α-SMA, were significantly reduced. These results indicate that fraxetin inhibits lipid peroxidation and has a protective effect on pulmonary fibrosis (Zhai et al., 2023).

### 4.4 Pulmonary hypertension

Pulmonary hypertension is the term used to describe a group of disorders characterized by abnormally high pressures in the pulmonary arteries (Poch and Mandel, 2021). For pulmonary hypertension, a low-salt diet, diuretics, and oxygen therapy are general management strategies. Pharmacotherapy includes calcium channel blockers or targeted prostacyclin, nitrate oxide, and endothelin pathways. There are also surgical therapies such as endarterectomy (Mocumbi et al., 2024). It has been demonstrated that lipid peroxidation causes vascular remodeling and PASMC proliferation, which in turn causes the onset and exacerbation of pulmonary hypertension. The generation and buildup of ROS are facilitated by hypoxia and pulmonary inflammatory factors, which can result in oxidative stress, elevated levels of lipid peroxidation, and the creation of hazardous metabolic chemicals, ultimately leading to pulmonary hypertension (Sharma et al., 2016). The main avenues for the use of natural ingredients in the treatment of pulmonary hypertension include raising or restoring the level of antioxidant enzymes, and inhibiting the pro-inflammatory NF-κB pathway.

Baicalein is a flavonoid extracted from the root of Scutellaria baicalensis (*Scutellaria baicalensis* Georgi). In previous studies, baicalein was shown to promote ROS attenuation by activating CAT (Liu et al., 2021). Rats were given 50 and 100 mg/kg/day of baicalein for 28 days. Both dosages demonstrated antioxidant action. The dosage of 100 mg/kg/day downregulated NF-κB expression, decreased MDA levels, enhanced GPx and SOD antioxidant activities (Shi et al., 2018).

Cyanidin-3-O-β-glucoside is a classical anthocyanin. It is widely found in numerous dark-colored foods, such as mulberry and black rice. Experiment proof that in the oxidation of soybean phosphatidylcholine liposomes, Cyanidin-3-O-β-glucoside efficiently scavenged the peroxyl radicals generated in the aqueous phase (Sousa et al., 2016). Pulmonary arterial hypertension rats were orally administered Cyanidin-3-O-β-glucoside, and it was found that ingestion of Cyanidin-3-O-β-glucoside increased SOD levels, reduced oxidative stress, and lipid peroxidation. Cyanidin-3-O-β-glucoside also plays a role in reversing vascular remodeling caused by monocrotaline (MCT) (Ouyang et al., 2021).

Resveratrol is a polyphenolic phytoalexin from the roots of Veratrum grandiflorum (*Veratrum grandiflorum* (Maxim. ex Miq.) O.Loes.), and it is particularly plentiful in fresh grape skin (Singh et al., 2019). In the exploration of hypoxic pulmonary hypertension treatment, rats were treated with resveratrol by gavage for 28 days, and found that it could reduce the ROS and H_2_O_2_ production, and decrease NF-κB expression (Xu et al., 2016). Carvacrol is one of the main components of Origanum vulgare (*Origanum vulgare* L.) and Thymus vulgaris (*Thymus vulgaris* L.) essential oil. *In vitro* test found that after 600 μM carvacrol treatment, the levels of antioxidant enzymes SOD and GSH were restored, and the hypoxia-mediated lipid peroxidation was significantly reduced. *In vivo* test found that 50 and 100 mg/kg carvacrol prevents hypoxia-induced right ventricular hypertrophy and pulmonary vascular remodeling. This indicates that carvacrol has an inhibitory effect on the oxidative damage of PASMCs under hypoxic conditions. In terms of related pathways, carvacrol suppresses the expression of procaspase-3 and promotes the activation of caspase-3, significantly inhibiting hypoxia-induced ERK1/2 and Akt phosphorylation (Zhang et al., 2016).

Arctigenin is a lignan from traditional Chinese medicine Arctium lappa (*Arctium lappa* L.). Rats were given arctigenin 50 mg/kg/day for 28 days intraperitoneally. The results demonstrated that arctigenin treatment significantly decreased MDA levels and elevated SOD activity, halted the progression of MCT-induced pulmonary arterial hypertension in rats by blocking oxidative stress and lipid peroxidation. In addition, arctigenin inhibited the MCT-induced elevation of NLRP3, caspase-1, and interleukin 1-β expression (Jiang et al., 2018).

Berberine is a yellow isoquinoline alkaloid present in various plants such as Berberis vulgaris (*Berberis vulgaris* L.), and its antioxidant activity in other tissues has been demonstrated. After 3 weeks of intraperitoneal injection of berberine, the antioxidant activity of SOD, GPx, and CAT in rats was effectively restored, and MDA levels in lung tissue were reduced. An optimal action concentration of 30 mg/kg was also determined. No experimental animals died in the berberine treatment group, while the mortality rate in the other groups was 57%, indicating that the treatment with berberine has certain efficacy and safety (Beik et al., 2023).

18β-Glycyrrhetinic Acid is a kind of pentacyclic triterpenes, is the main bioactive ingredient of Glycyrrhiza uralensis (*G. uralensis* Fisch. Ex DC.) root, and its antioxidant activity was observed. Researchers gave 18β-Glycyrrhetinic Acid to rats by oral administration at three doses for 21 days. MCT-treated rats showed a significant reduction of SOD, CAT, and GPX concentrations and increased MDA levels, which were reversed by 18β-Glycyrrhetinic Acid, and experimental data support the notion that 18β-Glycyrrhetinic Acid is beneficial in the treatment of pulmonary arterial hypertension (Zhang et al., 2019).

As a naturally occurring double cyclic sesquiterpene, free and nanoemulsified β-caryophyllene is isolated from plant essential oils. When administered β-caryophyllene to rats, researchers found that it can improve pulmonary hypertension markers and attenuate oxidative stress-induced lipid damage. It is thought to work by replenishing antioxidant enzymes and avoiding GSH deficiency. Crucially, it can reduce the production of ROS in vascular cells by inhibiting NADPH oxidase activity and xanthine oxidase protein expression (Carraro et al., 2024).

Crocin is the main pharmacologically active ingredient of Saffron (*C. sativus* L.), which is a water-soluble carotenoid. Crocin was administered intraperitoneally to MCT-induced pulmonary arterial hypertension rats. The study revealed that crocin affected the oxidation resistance 1 (OXR1) signal pathway in rats by regulating SOD, GSH and CAT, and had a protective effect on MCT-induced pulmonary hypertension (Dianat et al., 2020).

Diosgenin is a kind of steroid compounds that exist in nature. Rats were given diosgenin orally for 3 weeks, and the findings of the experiment showed that diosgenin normalized GSH while ameliorating myeloperoxidase activity and TNF-α levels. Diosgenin caused a decrease in mortality percentage reaching 9.09%, which illustrates the positive effects it may have on clinical application (Ahmed et al., 2014).

### 4.5 Chronic obstructive pulmonary disease

Through a number of processes, oxidative stress-lipid peroxidation exacerbates airway inflammation and causes tissue damage. Raised ROS levels and consequent lipid oxidative damage are two processes caused by environmental variables and lung inflammation (Dailah, 2022). MDA, a byproduct of lipid peroxidation, is the one that has been investigated the most. In 2004, Aldehyde concentration was first used as a biomarker for oxidative stress and lipid peroxidation in patients with chronic airway inflammation (Corradi et al., 2004). It was demonstrated that the MDA concentration in the sputum was further increased in COPD aggravation (Antus, 2016). Exposure to particulate air pollution in patients with COPD increases serum thromboxane levels and the risk of concurrent cardiovascular disease (Wang et al., 2022c). Currently, general treatments for COPD include the use of bronchodilators to improve smooth muscle tone and the improvement of airflow by suppressing inflammation. These methods are used to alleviate symptoms, improve lung function, and reduce the risk of exacerbations and death (Sandelowsky et al., 2021). Natural ingredients affect the level of lipid peroxidation and exert a therapeutic effect on COPD, mainly by regulating and restoring the level of antioxidant enzymes, and then reducing the production of toxic metabolites such as MDA, and there is a certain dose correlation in this process. While regulating the level of lipid peroxidation, natural ingredients also effectively inhibit inflammatory pathways (such as NF-κB, Nrf2), effectively alleviate and control the development of COPD, and have a positive effect on improving the quality of life of patients.

One of the natural sources of luteolin is an herb used in Chinese medicine called honeysuckle (*Lonicera japonica* Thunb.). Experiments proved that it could alleviate the oxidative stress in A549 cells, manifested by enhancing SOD activity and inhibiting the production of MDA and lactate dehydrogenase (Zhou et al., 2023).

Traditional Chinese medicine frequently uses citrus peel to treat lung conditions. Citrus peels are a natural source of hesperidin, a flavonoid that has anti-inflammatory and anti-oxidative stress properties as well as the ability to efficiently lower lipid peroxidation levels. Researchers intraperitoneally injected hesperidin into mice for 21 days. The result proved that it reduced the level of lipid peroxidation product MDA through increased SOD and CAT levels, which is associated with the SIRT1/PGC-1α/NF-κB signaling axis. In this experiment, budesonide (2 mg/kg) was used as the control group. The experimental results show that the number of dead cells in hesperidin-h group (50 mg/kg/d) was significantly lower than in budesonide group and hesperidin-l group. Hesperidin-h group’s expression promotion effect of SIRT1 and PGC-1α was better than that of hesperidin-l group. These results indicated that high-dose hesperidin had a stronger regulatory effect on oxidative stress and inflammatory response (Wang et al., 2020).

Gallic acid is a polyhydroxy phenolic compound, it extensively consists in the roots, stems, leaves, fruits, skins, flowers and seeds of many medicinal plants (Bai et al., 2021). The study found that for COPD exacerbation model mice, daily administration of gallic acid starting 7 days before elastase instillation through reduced production of ROS, restored the level of SOD and GSH, and reduced MDA. In the process of gallic acid action, it effectively prevented NF-κB activation, increased Nrf2 protein levels, and prevented COPD deterioration (Singla et al., 2021).

Mice were administered betulin, a triterpene substance produced from birch (*Betula lenta* L.) bark, for 8 weeks at 20 mg/kg and 40 mg/kg. The results indicate that betulin had the effect of raising blood SOD and CAT levels and decreasing MDA content. Both positive control and betulinol (40 mg/kg) showed good efficacy, but betulinol remained slightly worse for some indicators than dexamethasone (2 mg/kg). In this process, betulin inhibits the pro-inflammatory factors (such as TNF-α, IL-6, and IL-1β) and the ROCK/NF-κB pathway (Chunhua et al., 2017).

### 4.6 Lung cancer

In lung cancer, lipid peroxidation has two different functions. On the one hand, lowering lipid peroxidation and oxidative stress levels helps stop cancer cells from growing in situations when there is sporadic hypoxia. Conversely, aberrantly high levels of lipid peroxidation in cancer cells contribute to the promotion of cancer cell apoptosis. General treatments for lung cancer include surgery, targeted drug therapy, radical radiotherapy, and stereotactic ablative radiotherapy. In addition, immunotherapies are still under development (Hirsch et al., 2017; Neal et al., 2019). At present, promoting cancer cell death by increasing lipid peroxidation levels has been a hot topic in recent years. Some well-studied natural ingredients related to lung diseases have been shown to play a role in promoting lung cancer cell death. These natural products are diverse, including flavonoids, anthraquinones, phenolic compounds, alkaloids, peptides. Natural ingredients can regulate the level of lipid peroxidation and apoptosis-related signals such as PI3K/AKT, thus playing a more effective role in the intervention of lung cancer.

Luteolin is a flavonoid that plays a therapeutic role in a variety of lung-lineage diseases. The study on NCI-H460 and H1299 non-small cell lung cancer cells as well as xenograft model mice showed that luteolin is a radiosensitizer for non-small cell lung cancer. Luteolin enhances ROS damage and lipid peroxidation by activating the p38/ROS/caspase cascade (Cho et al., 2015).

Resveratrol is a polyphenolic compound considered as a strong antioxidant (Parsamanesh et al., 2021). Resveratrol was found to induce ROS production, impair SOD activity, disrupt the chemical-antioxidant system, and cause apoptotic cell death in A549 cells through oxidative stress (Gu et al., 2015).

Sinapine was shown to be selectively toxic to non-small cell lung cancer cells in cellular experiments. Both cell and animal studies have shown that sinapine treatment leads to abnormally elevated ROS. Then lipid peroxidation levels increased, thus inducing the death of lung cancer cells. During the experiment, there was no significant weight loss in the treatment group, indicating that sinapine has a certain safety (Shao et al., 2022). Dihydroartemisinin is the first-generation derivative of artemisinin, which is derived from the annual compositae family member Artemisia annua (*Artemisia annua* L.). Preclinical and clinical studies provide stronger evidence of its anticancer potential (Dai et al., 2021). Vitro experiments demonstrated that dihydroartemisinin deepens oxidative damage, in the process, intensified expression of COX-2 and reduced expression of GPX4 were observed. And then it induces lipid peroxidation accumulation. These changes lead to DNA damage and endoplasmic reticulum stress, and promote the immunogenic death of lung cancer cells. *In vivo* experiments, it was found that dihydroartemisinin (10 mg/kg b.w.) promoted the increase of ROS production in tumor tissues, accompanied by significant apoptosis of tumor cells. The use of doxorubicin in the positive control group further demonstrated that dihydroartemisinin has the characteristics of good inhibitory effect, selective inhibition of malignant cells, and less toxicity (Han et al., 2023).

Formosanin C is a diosgenin saponin from Paris polyphylla (*Paris yunnanensis* Franch.). Cellular experiments have shown that it causes excessive ROS production and GSH depletion, leading to oxidative stress, which in turn inhibits the growth of non-small cell lung cancer cells. *In vivo* experiments on mice allograft tumor models, Formosanin C exerted a good anti-tumor effect, and the inhibitory effect of Formosanin C (1 mg/kg) was better than that of the positive control ciplatin (1 mg/kg) (Li et al., 2023).

Purpurin is a naturally occurring anthraquinone identified from Rubia cordifolia (*Rubia cordifolia* L.) roots (Singh et al., 2021). Purpurin was tested on A549 cells, and it was found that purpurin promotes cell death by promoting ROS-mediated oxidative stress and lipid peroxidation. This is manifested by the elevation of MDA and depletion of intracellular GSH in a time-dependent manner. The PI3K/AKT cascade signaling pathway is also modulated in this process (Bo et al., 2021).

## 5 Discussion

Pulmonary diseases seriously endanger people’s health due to high morbidity and mortality. Currently, most effective treatments for pulmonary diseases have side effects that make it difficult to meet existing treatment needs. For example, glucocorticoids such as dexamethasone are commonly used in the respiratory system, but they have many side effects that can cause headaches, vomiting, gastrointestinal bleeding, osteoporosis, and more (RECOVERY Collaborative Group et al., 2021; Nathan et al., 2023; Snow et al., 2023). Therefore, there is an urgent need to develop effective, low-side-effect, inexpensive drugs. Lipid peroxidation is a crucial feature of the novel form of cell death-ferroptosis, which plays an important role in the development of lung system diseases. Inhibiting lipid peroxidation in normal cells can improve disease prognosis while inducing lipid peroxidation in cancer cells can alleviate cell proliferation and migration. To emphasize the importance of the lipid peroxidation mechanism itself, this article reviews the relevant literature on the treatment of pulmonary diseases by natural ingredients interfering with lipid peroxidation, and briefly introduces the experimental process and mechanism. Natural ingredients regulate lipid peroxidation through various targets and pathways, mainly including the System X_c_^−^, Nrf2/GPX4, and Nrf2/HO-1. In addition, natural ingredients have antioxidant properties, which can directly or indirectly eliminate ROS, alleviate oxidative damage, and provide a promising approach for preventing and treating respiratory diseases. In ALI, the Nrf2/HO-1 pathway is the core mechanism of targeted lipid peroxidation therapy. Natural ingredients can effectively alleviate changes related to oxidative stress by upregulating the expression of the Nrf2 pathway and promoting the expression of downstream antioxidant enzymes, thereby reducing lung inflammation. The treatment of asthma by regulating lipid peroxidation through natural ingredients is mainly achieved by enhancing the antioxidant system and reducing inflammation of tracheal epithelial cells. In this process, it may also involve pathways such as PI3K/AKT, ERK/JNK, Nrf2, etc. In pulmonary fibrosis, natural ingredients can reduce the induction of pro-fibrotic factors such as TGF-β and fibronectin by clearing lipid peroxides, thus delaying the progression of pulmonary fibrosis. The main methods of using natural ingredients to treat pulmonary arterial hypertension are inhibiting the pro-inflammatory NF-κB pathway, and increasing or restoring antioxidant enzyme levels, thereby reducing lipid peroxidation levels. The therapeutic effect on COPD is mainly achieved by effectively inhibiting inflammatory pathways such as NF-κB and Nrf2, thus reducing the production of toxic metabolites such as MDA. The treatment of lung tumors mainly involves enhancing lipid peroxidation to promote cancer cell death, as well as regulating apoptosis-related signals such as PI3K/AKT.

Although the use of natural ingredients for the prevention and treatment of respiratory diseases has broad prospects, there are still some limitations that need to be considered. Firstly, in the pathogenesis of respiratory diseases, lipid peroxidation is one of the important features of ferroptosis. Detailed studies are needed on iron metabolism, lipid peroxidation, and subcellular structure to distinguish the correlation between different stages of ferroptosis and respiratory diseases. Lipid peroxidation, as an important pathophysiological process, has also not been fully elucidated in its detailed mechanisms (Hu et al., 2024). Multi-omics technologies, including genomics, transcriptomics, proteomics, and metabolomics, may provide us with the opportunity to deeply understand these complex processes (Kong et al., 2024; Wang et al., 2024). In addition, organoid modeling and spatial transcriptomics techniques provide powerful tools to study the spatial specificity of disease development. For example, human lung organoids have been established as highly transferrable three-dimensional *in vitro* model systems for lung research in recent years, which have opened possibilities for precise *in vitro* research and a deeper understanding of mechanisms underlying lung injury and regeneration (Kühl et al., 2023; Thangam et al., 2024). Through these tools, we can more precisely locate the key links of lipid peroxidation in lung diseases and explore how natural ingredients can act by regulating these links.

Secondly, botanical drugs, as an important part of traditional medicine, have a long history and rich experience in treating lung diseases (Yuhao et al., 2024). However, the active ingredients of botanical drugs are often unclear, which may limit their application and development in modern medicine. To fully utilize the potential of natural ingredients, we need to further explore their active ingredients and mechanisms of action, determine the optimal administration method and frequency of different drugs. Through modern separation and extraction techniques and bioactivity screening techniques, we can gradually clarify the active ingredients in botanical drugs and reveal their molecular mechanisms in treating lung diseases by regulating lipid peroxidation (Wawoczny and Gillner, 2023; Fan et al., 2020).

Thirdly, some preliminary results have been achieved in the experiments of natural ingredients in treating lung diseases by regulating lipid peroxidation (Wang et al., 2021; Wang et al., 2022d). For example, a number of antioxidant drugs (such as nobiletin, green tea extract) have been shown to reduce lung inflammation and injury and improve lung function in patients (Qin et al., 2024; Veerman et al., 2022; Albrecht et al., 2023). However, most of these studies are still in the early stages and more clinical trials are needed to verify their efficacy and safety. Meanwhile, the development of novel drugs targeting lipid peroxidation is also underway (Azmi et al., 2019). For example, a number of small-molecule drugs targeting lipid metabolic pathways are being developed for the treatment of lung diseases (Wang et al., 2022a; Ma et al., 2024). These novel drugs are expected to provide new options for the treatment of lung diseases.

This paper mainly summarises the mechanisms and results of natural ingredients interfering with lipid peroxidation for the treatment of respiratory diseases, with fewer studies on their adverse reactions and side effects. Meanwhile, the results of pharmacokinetic and clinical experimental studies are lacking. Follow-up studies may be interpreted to address the above issues. Besides, the extraction and processing of the drug and its combination with modern nanotechnology and so on is also a new direction (Lalsangpuii et al., 2024). Furthermore, natural ingredients not only treat respiratory diseases by regulating lipid peroxidation, but also include mechanisms such as inflammation, apoptosis, and cell proliferation. Therefore, the combined effect of multiple mechanisms is also worth studying (Dong et al., 2024; Irshad et al., 2022). As basic and clinical research continues to progress, it is becoming increasingly feasible to incorporate targeting lipid peroxidation modulation into clinical practice for the prevention and treatment of respiratory diseases.

## 6 Conclusion

In summary, many natural ingredients have shown therapeutic effects on pulmonary diseases by interfering with lipid peroxidation. However, there are many shortcomings in the current research. Although natural ingredients are empirically used in many areas for the treatment of respiratory diseases, it is clear that the clinical promotion of natural ingredients would benefit from more in-depth studies on their mechanisms of action. It is believed that better use of natural ingredients, which are highly practical, inexpensive, and widely available, will add new and powerful tools to the treatment of respiratory diseases.
